# Retroperitoneal ectopic pregnancy: a case report and review of the literature

**DOI:** 10.1186/s12884-017-1542-y

**Published:** 2017-10-16

**Authors:** Man Yang, Lamu Cidan, Dan Zhang

**Affiliations:** Section of Reproductive Endocrinology, Department of Obstetrics and Gynecology, West China Second University Hospital, Sichuan University, No. 20 Section 3 of South Renmin Road, Chengdu, Sichuan Province 610041 China

**Keywords:** Case report, Ectopic pregnancy, Retroperitoneal ectopic pregnancy, Laparoscopy

## Abstract

**Background:**

Retroperitoneal ectopic pregnancy (REP) is an extremely rare type of ectopic pregnancy, with a total of less than 20 cases reported in the English literature. However, failure to recognize REP may result in severe consequences.

**Case presentation:**

We report a case of 32-year-old woman with REP. She had amenorrhea, left lower abdominal pain, but no vaginal bleeding. Her urine human chorionic gonadotropin (HCG) test was positive and blood HCG level was 1880 m-international units per milliliter (mIU/mL). Transvaginal ultrasound sonography showed a left adnexal mass. Laparoscopy found an enlarged uterus, normal right uterine tube and ovary, and normal left uterine tube. The left ovary was partly covered by a blood clot, but appeared normal after removing the clot. There was a 10-mm circular peritoneal defect located lateral to the left sacrocervical ligament, anterior to the left ovarian fossa, and next to the lower edge of the left broad ligament. The patient was diagnosed of having REP with the gestational tissues covered by the peritoneum. The REP was removed by laparoscopic surgery. Bleeding was stopped by bipolar coagulation and absorbable hemostatic cellulose. The patient recovered smoothly and was discharged on the next day after surgery. Her blood HCG returned to normal range 29 days after surgery.

**Conclusions:**

REP is very rare, but in any suspected case of ectopic pregnancy, caution must be paid to find signs of REP when the common sites of ectopic pregnancy do not have any positive findings.

## Background

Ectopic pregnancy is defined as the fertilized ovum implants in the tissues outside the endometrium within the body of the uterus. Although the incidence of ectopic pregnancy has increased during the past decades, the maternal morbidity and mortality of ectopic pregnancy have decreased due to improvement in early diagnosis using high-resolution transvaginal ultrasound sonography (TVS) and laparoscopy [1]. Retroperitoneal ectopic pregnancy (REP) is an extremely rare type of ectopic pregnancy. Jiang et al. [2] reviewed the English literature and found a total of 14 case reports of REP. Hall et al. [3] reported a case of REP in 1973 and Peter C. Sotus [4] reported another similar case in 1977. Both ectopic pregnancies were found in the retroperitoneal space located between the left side of the aorta and the superolateral side of the left iliac artery, with the placenta adhered to the psoas fascia [4]. It was postulated that the trophoblast invaded and penetrated through the peritoneum during the first few days of implantation, and then the peritoneum regenerated to cover the gestational tissues [4]. Plaus [5] reported a case of ectopic choriocarcinoma in the right kidney. Dmowski et al. [6] reported a case of REP in the head of pancreas. Lee et al. [7] reported a case of REP located in the left paraaortic region below the left kidney. Iwama et al. [8] reported a case of REP located over the aorta and inferior vena cava. Okorie [9] reported a cased of REP located similarly over the aorta and inferior vena cava near the second and third parts of the duodenum. Persson et al. [10] reported a case of REP located in the right obturator fossa. Martinez-Varea et al. [11] reported a case of REP located next to the left uterosacral ligament. Liang et al. [12] reported a case of REP located next to the abdominal aorta, ovarian vessels and the left renal vein. Here we report a case of REP located lateral to the left sacrocervical ligament, anterior to the left ovarian fossa, and next to the lower edge of the left broad ligament.

## Case presentation

**Fig. 1:**
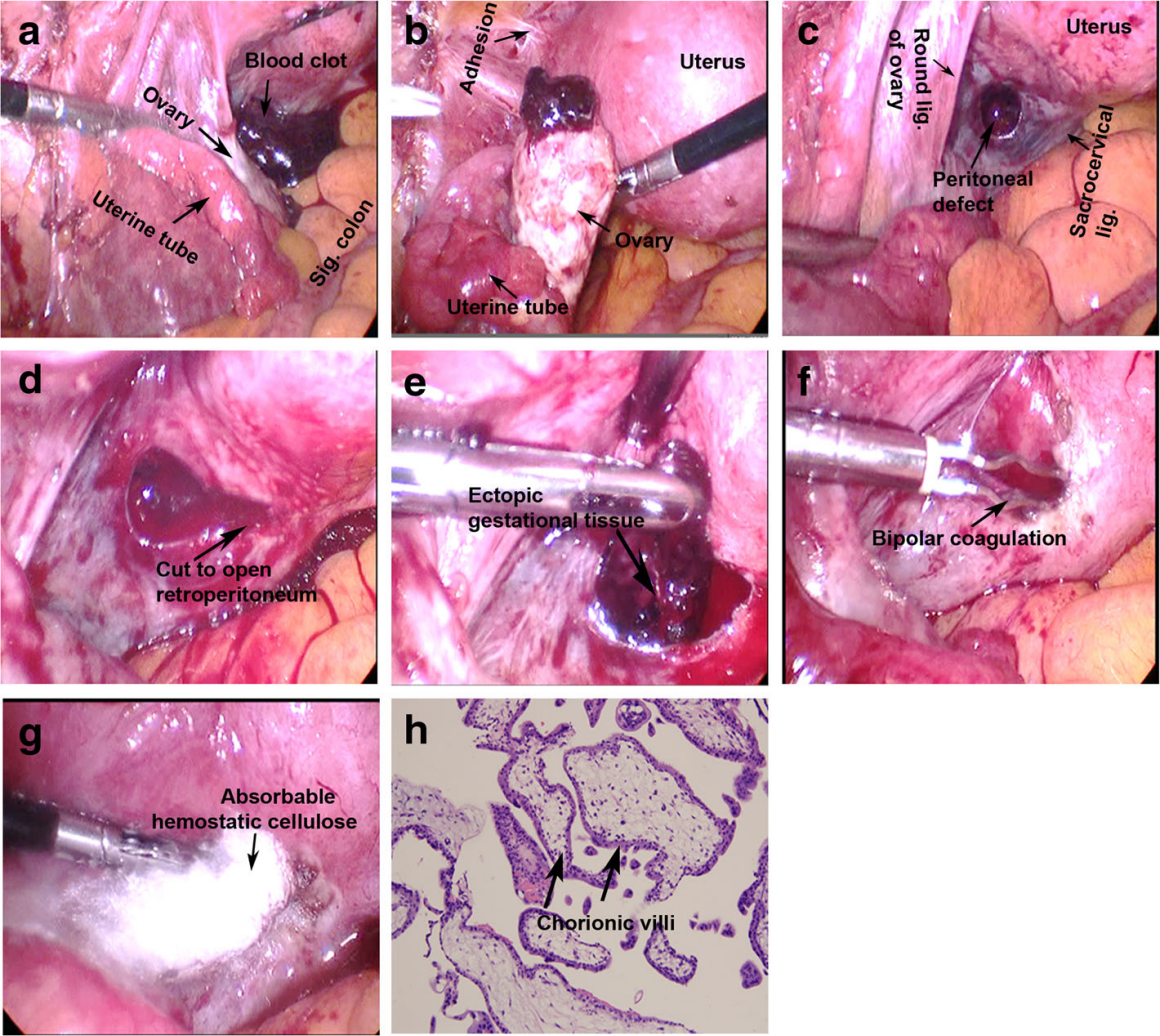
Photographs taken during laparoscopic surgery (**a** to **g**) and histopathology of the removed gestational tissues (**h**)

A 32-year-old woman, gravida 5, para 1 (12 years ago by Cesarean section) with 4 induced abortions (the last one 2 years ago), was admitted due to amenorrhea for 38 days and left lower abdominal pain for 2 days accompanied with mild nausea, tender breasts, and rectal pressure. Eight days prior to admission, her urine human chorionic gonadotropin (HCG) test was positive and blood HCG level was 1880 m-international units per milliliter (mIU/mL). There was no vaginal bleeding. Color TVS showed no intrauterine gestational sac, but there was a left adnexal heterogeneous mass approximately 21 mm × 14 mm × 20 mm in size with signs of blood supply. The right adnexa appeared normal. There was free fluid 38 mm in depth within the pouch of Douglas. The past and family histories were unremarkable. General physical examination revealed nothing remarkable and the vital signs were normal. Gynecological examination found that the uterine cervix was smooth with tenderness upon palpation and movement; the uterine body was enlarged equivalent to the size of 40-day-gestation, soft, and with smooth surfaces; the left adnexa was slightly thickened with tenderness; and the right adnexa was unremarkable. The patient was diagnosed as ectopic pregnancy and was prepared for emergent laparoscopy. Under laparoscopy, the uterus appeared enlarged equivalent to the size of 40-day-gestation, with smooth surfaces, but without bleeding; the right uterine tube and ovary appeared normal; the left uterine tube was normal (Fig. 1a); the left ovary was slightly enlarged, partly covered by a blood clot (Fig. 1a-1b), but had no rupture or bleeding after the clot was removed; there was a small amount of unclotted blood in the pouch of Douglas; there were no abnormal findings in the greater omentum, small intestines, colons, and mesenteries. After removing the blood clot and free blood, a circular peritoneal defect of approximately 10 mm in diameter was found lateral to the left sacrocervical ligament, anterior to the left ovarian fossa, and next to the lower edge of the left broad ligament (Fig. 1c). The surrounding peritoneum appeared bluish in color (Fig. 1c) and bled when pressured with a probe. A 10-mm cut was made medial to the defect to open into the retroperitoneal space (Fig. 1d). The blood clot with gestational tissues was pulled out of the retroperitoneal space (Fig. 1e). The retroperitoneal space was cleaned and irrigated with saline, where part of the left ureter was seen with edema. Bleeding was stopped by bipolar coagulation (Fig. 1f). Absorbable hemostatic cellulose (Surgicel® Fibrillar™ Hemostat, Ethicon, LLC, San Lorenzo, Puerto Rico, USA) was inserted into the retroperitoneal space to prevent further bleeding (Fig. 1g). Pathological examination of the gestational tissues found chorionic villi (Fig. 1h). The patient lost about 300 mL of blood during the surgery, but did not need any blood transfusion. She recovered smoothly and was discharged on the next day after surgery. The patient’s blood HCG level decreased to 320 mIU/mL 1 week after surgery and returned to normal range 29 days after surgery.

## Discussion

Ectopic pregnancy occurs in approximately 2% of all pregnancies. Over 95% of ectopic pregnancies occur in the uterine tube (also called Fallopian tube). The pathogenesis is still not clear. The known risk factors include reduced or impaired tubal transport activity, increased tubal receptivity for blastocyst implantation, tubal damage due to surgery or infection, in vitro fertilization, and cigarette smoking [13, 14]. REP is very rare with a total of less than 20 cases reported in the English literature [2]. Seven of the REP cases occurred in the patients under in vitro fertilization and embryo transfer. Two mechanisms were hypothesized: spontaneous retrograde migration of the embryo after intrauterine transfer and uterine perforation with unintended retroperitoneal placement at the time of transfer [7]. In our case, the patient did not receive any embryo transfer. She did not intend to be pregnant, so the ectopic pregnancy was an unintended spontaneous conception. However, the patient had a Cesarean section 12 years ago and 4 times of induced abortions with the last abortion occurred 2 years ago, which might have caused pelvic infection and/or inflammation that is evidenced by the adhesion of pelvic peritoneum (Fig. 1b). The adhesive changes in the pelvis including the uterine tubes might be a possible cause of the ectopic pregnancy. The patient did not display any associated risk factors such as cigarette smoking, multiple partners, or age at first intercourse. The exact reason for this REP is not clear. The circular peritoneal defect (Fig. 1c) might be the location where the blastocyst originally implantated, and then the peritoneum regenerated to cover most of it except at this defect according to Dr. Sotus’s hypothesis [4]. On the other hand, the defect could be caused by that the once covered gestational tissues eroded and ruptured the peritoneum. Based on the observation that the gestational tissues were completely covered by the retroperitoneum, there is no doubt that the ectopic pregnancy was a REP. The location of the current REP was similar to the REP reported by Martinez-Varea et al. in 2011 [11], except that the previous case had no peritoneal defect, thus the gestational tissues were completely covered by the peritoneum. Nevertheless, one important lesson from this case is that whenever there is any suspected ectopic pregnancy, it is absolutely necessary to rule out any possibility of REP. Particularly, for the current case, there was a peritoneal defect covered by a blood clot, which could lead the physician to believe that the ectopic pregnancy was attached over the peritoneum and had already detached (thus aborted). But in fact, the ectopic pregnancy was hiding behind the peritoneum. Failure to remove the gestational tissues might allow them to continue growth and invasion into the ureter and branches of internal iliac vessels, hence resulting in severe consequences.

## Conclusions

REP is very rare, but in any suspected case of ectopic pregnancy, caution must be paid to find signs of REP when the common sites of ectopic pregnancy do not have any positive findings.

## Abbreviations

HCG: Human chorionic gonadotropin
REP: Retroperitoneal ectopic pregnancy
TVS: Transvaginal ultrasound sonography
